# Effects of Body Weight Unloading and Treadmill Belt Surface on Tibial Acceleration-Based Loading Indicators During Running on a Microgravity Treadmill

**DOI:** 10.3390/jcm15103654

**Published:** 2026-05-09

**Authors:** Łukasz Oleksy, Anna Mika, Martyna Sopa, Artur Stolarczyk, Olga Adamska, Maciej Kuchciak, Miłosz Szczudło, Paulina Ciepiela, Rafał Buryta, Paweł Reichert, Renata Kielnar

**Affiliations:** 1 Department of Orthopedics, Traumatology and Hand Surgery, Faculty of Medicine, Wroclaw Medical University, 50-556 Wroclaw, Poland; loleksy@oleksy-fizjoterapia.pl (Ł.O.); pawel.reichert@umw.edu.pl (P.R.); 2 Institute of Physical Culture Sciences, University of Szczecin, 71-065 Szczecin, Poland; rafal.buryta@usz.edu.pl; 3 Oleksy Medical & Sport Sciences, 37-100 Lancut, Poland; 4 Institute of Clinical Rehabilitation, University of Physical Culture in Kraków, 31-571 Krakow, Poland; 5 Institute of Applied Mechanics, Faculty of Mechanical Engineering, Poznan University of Technology, 61-138 Poznan, Poland; martyna.sopa@put.poznan.pl; 6 Department of Orthopaedics and Rehabilitation, Medical and Dentistry Faculty, Medical University of Warsaw, 02-091 Warsaw, Poland; artur.stolarczyk@wum.edu.pl; 7 Department of Ophthalmology, Faculty of Medicine, Collegium Medicum Cardinal Stefan Wyszyński University in Warsaw, 01-815 Warsaw, Poland; o.adamska@uksw.edu.pl; 8 Faculty of Physical Culture Sciences, Collegium Medicum, University of Rzeszów, 35-959 Rzeszow, Poland; mkuchciak@ur.edu.pl (M.K.); mszczudlo@ur.edu.pl (M.S.); 9 University Center for Research and Development in Health Sciences, Faculty of Health Sciences and Psychology, Institute of Physiotherapy, Collegium Medicum, University of Rzeszów, 35-315 Rzeszow, Poland; pciepiela@ur.edu.pl (P.C.); kielnarrenata@o2.pl (R.K.)

**Keywords:** microgravity treadmill, body weight support, tibial stress fractures, running biomechanics, treadmill running, bone loading

## Abstract

**Background/Objectives**: The aim of this study was to compare tibial acceleration and acceleration-based indicators associated with tibial loading during running under full body weight (BW) conditions and with body weight support (80%, 60%, and 40% BW) on a microgravity treadmill, and to examine the effect of treadmill surface by comparison with a conventional treadmill. **Methods**: Twenty-six healthy, physically active adults (age 18–40 years) completed running trials at a constant speed of 8 km/h. Tibial acceleration was recorded using inertial measurement units. **Results**: Cadence decreased significantly with increasing levels of body weight support, while no differences were observed between a conventional treadmill running and microgravity treadmill running at 100% BW. Axial acceleration parameters demonstrated a consistent reduction in acceleration magnitude associated with progressive unloading. The magnitude of axial acceleration (MAA), as well as peak positive (PPAA) and negative (PNAA) axial accelerations, decreased significantly, with the largest reductions observed at 60% and 40% BW. Temporal parameters showed a different pattern: time to peak positive axial acceleration increased with unloading, indicating delayed peak loading. The duration of positive axial acceleration exhibited a non-linear response, increasing at moderate unloading levels (80% and 60% BW) and decreasing at 40% BW. In contrast, the duration of negative axial acceleration did not change significantly, although overall contact time increased with unloading. **Conclusions**: The findings of this study indicate that progressive body weight unloading during running on a microgravity treadmill leads to a significant reduction in impact-related tibial acceleration indicators associated with mechanical loading exposure, particularly in parameters describing the amplitude and dynamics of axial accelerations. Running at 100% BW on both treadmill systems demonstrated overall biomechanical similarity; however, several side-specific differences in tibial acceleration parameters suggest that treadmill surface and system characteristics may exert a subtle influence on local acceleration measurements. Moderate unloading (~80% BW) may provide an optimal balance between load reduction and preservation of natural running mechanics.

## 1. Introduction

Overuse injuries of the lower extremities, including tibial stress fractures and overload-related changes within the knee joint, constitute a significant problem in physically active populations, particularly among adolescents and young athletes during periods of rapid growth. Their etiology is multifactorial and involves biomechanical, training-related, and environmental factors [1,2]. The pathogenesis of these injuries is closely associated with the accumulation of repetitive mechanical loads that exceed the adaptive capacity of bone tissue, leading to microdamage and, consequently, stress fractures [3,4].

The literature emphasizes that during running, the tibia is subjected to repetitive impact loads that may lead to microdamage and, ultimately, stress fractures [4,5]. Accelerations recorded at the tibial level are considered an indirect indicator of impact-related mechanical loading exposure acting on bone tissue, and higher values are associated with an increased risk of overuse injuries [5,6,7]. At the same time, it has been demonstrated that the magnitude of these loads depends on multiple factors, such as running speed, technique, body mass, and environmental conditions, highlighting their complex nature and the difficulty of their precise control [7,8]. Increases in running speed, surface incline, or additional external load lead to a significant rise in these loads [7,9]. Factors related to running technique and individual biomechanical properties also play a crucial role—individuals with a history of stress fractures exhibit higher ground reaction forces and tibial accelerations [4,10,11], while lower limb malalignment and impaired neuromuscular control further increase the risk of overload [2,11]. In this context, reducing mechanical loading exposure constitutes the cornerstone of treatment for stress fractures and other overload-related conditions, particularly in the tibia, where non-operative management remains the standard [5,12,13]. Contemporary guidelines indicate that treatment involves temporary reduction or complete cessation of pain-provoking activity, followed by a gradual return to loading based on clinical and imaging criteria [4,5]. This process is typically prolonged—the time to return to sport may range from several weeks to as long as 6–27 weeks, and in collegiate athletes averages approximately 12–13 weeks [12]. Depending on the location and severity of the lesion, treatment may include both activity restriction and periods of complete unloading, followed by progressive reloading, which is associated with a substantial period away from full training and competition [5,12,13].

For this reason, methods that allow the maintenance of physical activity while reducing mechanical loads are of particular importance. In clinical practice, low-load forms of exercise such as swimming, stationary cycling, or partial weight-bearing exercises are commonly used to maintain fitness and limit muscle atrophy; however, they do not fully replicate the specificity of running [5,13]. In this context, increasing attention has been given to microgravity treadmills utilizing lower-body positive pressure (LBPP) technology, which enable controlled reduction in effective body weight and, consequently, decreased loading of the lower extremities [14,15,16]. Previous studies have shown that partial body weight support leads to reduced ground reaction forces and alterations in spatiotemporal parameters of running, such as increased contact time and decreased cadence [16,17,18]. At the same time, it has been demonstrated that the reduction in loading is not always linear, as the body may adaptively modify locomotor strategies, for example by changing lower limb stiffness or movement patterns [14]. This suggests that the actual reduction in loads acting on bone tissue may be partially compensated for by biomechanical adaptations. Despite growing interest in microgravity treadmills, the number of studies analyzing their effects on local tibial acceleration and acceleration-based indicators of loading remain limited. Most available studies focus on global biomechanical parameters, such as ground reaction forces, kinematic variables, or energy cost, whereas direct assessment of tibial acceleration as an indicator of impact loading is relatively scarce [14,15,16,17,18]. Additionally, existing studies employ heterogeneous measurement protocols, including different sensor locations, device types, and experimental conditions, which complicates comparisons between results.

Another important yet still poorly understood factor is the influence of surface type on the magnitude of loads generated during running. The literature indicates that changes in surface mechanical properties may affect running biomechanics; however, these effects are not consistent, as runners may biomechanically adapt and partially compensate for changes in surface stiffness [7,14,18]. In particular, there is a lack of studies examining the combined effects of body weight support and treadmill surface properties on local bone loading. Therefore, there is a need for studies evaluating the influence of unloading level and surface type on tibial acceleration characteristics associated with mechanical loading exposure, taking into account local biomechanical indicators. It is particularly important to determine whether load reduction is linear with increasing levels of unloading or whether it is modified by adaptive changes in movement strategy. Importantly, microgravity treadmills differ from conventional treadmills not only in the ability to manipulate effective body weight, but also in belt structure, compliance, and lower-body positive-pressure system characteristics. Therefore, comparing running at 100% BW between treadmill systems is necessary to determine whether changes in tibial acceleration observed during unloading are attributable primarily to body-weight support itself or are partially influenced by treadmill-system mechanics.

The aim of the present study was to compare the magnitude of loads acting on the tibia during running under full body weight (BW) conditions and with body weight support at 80% BW, 60% BW, and 40% BW on a microgravity treadmill. Additionally, the effect of treadmill surface type was analyzed by comparing the cushioned surface used in a microgravity treadmill with that of a conventional treadmill. This study attempts to address an existing research gap by providing data on local indicators of bone loading, which are relevant from both clinical and training perspectives.

## 2. Materials and Methods

### 2.1. Study Participants

A total of 26 healthy (12 male and 14 female), physically active adults aged between 18 and 40 years (body mass 62.1 ± 10.5 kg; height 1.72 ± 0.2 m; Mean ± SD) participated in the study. All participants were healthy and physically active adults. “Physically active” was defined as participation in regular physical activity at least 3 times per week, accumulating a minimum of 150 min of moderate-to-vigorous physical activity per week during the preceding 6 months. Participants were not recruited based on competitive running status. Inclusion criteria required participants to be free from lower limb pain, injury, or disease within the previous 12 months and without any systemic illness. Additionally, none of the participants had a history of tibial stress injury. All participants provided written informed consent prior to participation. The study protocol was approved by the Ethics Committee at the Regional Medical Chamber in Kraków (No. 35/KBL/OIL/2024) and conducted in accordance with the Declaration of Helsinki (1964) and its subsequent amendments.

### 2.2. Study Design

A cross-sectional study design was used to investigate the relationship between tibial acceleration, the level of body weight support (BWS), as well as running surface (microgravity vs. conventional treadmill). Each participant attended a single laboratory testing session. All measurements were performed by the same team of experienced researchers. Body mass and height were recorded prior to testing. Participants completed a standardized 10 min warm-up before the measurements. Both lower limbs were included in the analysis.

### 2.3. Instrumentation and Data Acquisition

Tibial acceleration was recorded using the Delsys Trigno Avanti™ wireless system (Delsys Inc., Natick, MA, USA), equipped with 9-degree-of-freedom inertial measurement units (IMUs), comprising tri-axial accelerometers (±16 g, 16-bit resolution), tri-axial gyroscopes, and tri-axial magnetometers. Sensors were bilaterally placed over the tibial tuberosity according to the manufacturer’s guidelines and secured using straps and additional elastic tape to minimize soft tissue artifacts. Acceleration data were sampled at 148 Hz, which corresponds to the standard IMU acquisition mode for the system. All signals were transmitted wirelessly and recorded using proprietary Trigno Discover 2.0 software (Delsys Inc., Natick, MA, USA, and then exported for further processing in MATLAB R2025a (The MathWorks, Natick, MA, USA).

### 2.4. Experimental Procedure

Participants performed running trials on two treadmill systems:

- a microgravity treadmill (BOOST, Woodway, Waukesha, WI, USA)
- a conventional motorized treadmill (Cosmed, Rome, Italy).

The microgravity treadmill uses lower-body positive pressure (LBPP) to reduce effective body weight via a pressurized air chamber. Participants wore specialized neoprene shorts and were sealed into the chamber. The chamber height was aligned with the iliac crest and calibrated according to manufacturer guidelines. Participants wore their own running shoes and were instructed not to modify their natural running technique. No instructions regarding foot strike pattern or cadence were provided to avoid altering habitual biomechanics.

### 2.5. Running Protocol

The running protocol on the microgravity treadmill consisted of four consecutive 1 min runs at a constant speed of 8 km/h, performed at 100%, 80%, 60%, and 40% of body weight (BW). Between each stage, participants walked for 1 min at 4 km/h, during which the level of body weight support was adjusted to the next condition. The order of unloading conditions was not randomized to minimize the time required for adaptation and to reduce potential disturbances in running mechanics associated with abrupt changes in loading. Importantly, all microgravity treadmill conditions were completed first as a continuous sequence. After completing the microgravity treadmill trials, participants rested for 5 min. Only one subsequent trial was then performed on the conventional treadmill, consisting of a 1 min run at 8 km/h. Tibial acceleration data were recorded continuously during all trials. Data from each 1 min running stage were included in the analysis.

### 2.6. Data Analysis

The data obtained from the system included triaxial linear acceleration (expressed as multiples of gravitational acceleration [g]) and triaxial angular velocity [°/s]. The Delsys Trigno sensors are inertial measurement units (IMUs) that record signals independently along three orthogonal axes (x, y, z), reflecting the three-dimensional motion of the tibial segment. Acceleration values were expressed in multiples of gravitational acceleration (1 g = 9.81 m/s^2^). The signal sampling frequency provided by the system was 148 Hz, and the treadmill belt speed was set to 8 km/h. All data were filtered using a fourth-order low-pass Butterworth filter with a cutoff frequency of 20 Hz. To align multiple acceleration and angular velocity waveforms, all time-dependent variables were segmented into steps. For each lower limb, a step was defined from the initial heel strike to the subsequent heel strike of the same limb. Heel strike events were identified based on characteristic peaks in the acceleration signal. These recordings were then normalized to 100% of the gait cycle. The segmented variables were subsequently averaged to generate a mean waveform, which was used for further parameter calculations [7,19]. On average, each 1 min trial included approximately 80–90 steps per participant, depending on individual cadence. All detected steps were included in the analysis. No steps were excluded unless clear signal artifacts or detection errors were identified. Data processing was performed in MATLAB R2025a (The MathWorks, Natick, MA, USA).

### 2.7. Variables

To assess running characteristics, the following parameters were calculated for both the right and left tibia [7,19]:

**Spatiotemporal parameter**

Cadence (steps/min)—The number of steps performed per minute, reflecting step frequency and running rhythm.

**Axial acceleration parameters (impact-related)**

Magnitude of Axial Acceleration (MAA) [g]—The difference between the minimum and maximum axial acceleration within a gait cycle. This parameter reflects the range of loading experienced by the tibia.

Peak Positive Axial Acceleration (PPAA) [g]—The maximal positive acceleration along the tibial axis associated with initial ground contact (impact peak).

Peak Negative Axial Acceleration (PNAA) [g]—The maximal negative axial acceleration related to the propulsion phase (push-off).

Time to Peak Positive Axial Acceleration (TPPAA) [ms]—The time from initial contact to the occurrence of the peak positive axial acceleration, expressed in milliseconds of the gait cycle. This parameter reflects temporal characteristics of impact loading.

Duration of Positive Axial Acceleration (DPPA) [%]—The duration during which axial acceleration remains positive within the gait cycle, reflecting the loading phase.

Duration of Negative Axial Acceleration (DPNA) [%]—The duration of negative axial acceleration within the gait cycle, associated with the propulsion phase.

**Anterior–posterior acceleration parameters (braking & propulsion)**

Peak Positive Anterior–Posterior Acceleration (PPAPA) [g]—The maximal forward-directed acceleration, associated with propulsive forces during running.

Peak Negative Anterior–Posterior Acceleration (PNAPA) [g]—The maximal backward-directed acceleration, reflecting braking forces during initial contact.

**Global loading parameter**

Maximal Resultant Acceleration (MRA) [g]—The maximum value of the resultant acceleration vector, calculated from axial, anterior–posterior, and medio-lateral components. This parameter represents the overall resultant acceleration acting on the tibial segment and should be interpreted as an indirect biomechanical indicator associated with mechanical loading exposure rather than a direct measure of bone stress.

### 2.8. Statistical Analysis

The normality of data distribution was verified using the Shapiro–Wilk test. To assess the effects of body weight support and body side, a two-way repeated-measures ANOVA was performed with BMS level (100%, 80%, 60%, 40% BW) and Side (left vs. right) as within-subject factors. For comparisons between the conventional treadmill and the 100% BW condition on the microgravity treadmill, a repeated-measures ANOVA including treadmill condition (conventional vs. microgravity treadmill 100% BW) and Side as factors was used. When significant effects were identified, post hoc comparisons were conducted using the Tukey test. Statistical significance was set at *p* < 0.05. Effect sizes (Cohen’s d) were calculated to estimate the magnitude of observed differences and were interpreted as small (0.2–0.3), medium (0.5), and large (>0.8) [20]. A priori power analysis indicated that a minimum of 25 participants was required to achieve a statistical power of 0.80 at an alpha level of 0.05. All analyses were performed using STATISTICA 13.0 (TIBCO Software Inc., Palo Alto, CA, USA).

## 3. Results

### 3.1. Spatiotemporal Parameter

As the level of body weight support increased during running on the microgravity treadmill, cadence decreased significantly (Table 1).

### 3.2. Axial Acceleration Parameters

Analysis of axial acceleration parameters revealed a consistent and progressive reduction in acceleration magnitude with increasing levels of body weight support. The most pronounced changes were observed in variables describing the amplitude of acceleration. The magnitude of axial acceleration (MAA) decreased systematically across conditions, with the highest values recorded during running at 100% BW and the lowest values at 40% BW. This reduction was significant and characterized by a very large effect size, indicating a strong influence of unloading on axial acceleration variability (Table 2).

A similar pattern was observed for peak positive axial acceleration (PPAA) (Table 3) and peak negative axial acceleration (PNAA) (Table 4), both of which showed significant reductions with progressive unloading. The decrease in PNAA was particularly marked, suggesting a substantial attenuation of impact-related acceleration during the push-off phase.

Temporal parameters demonstrated a different response pattern compared to amplitude-based variables. The time to peak positive axial acceleration (TPPAA) increased with progressive unloading, indicating a delayed occurrence of peak loading. This effect was statistically significant, particularly for the right limb, with a large effect size (Table 5).

The duration of positive axial acceleration (DPPA) (Table 6) showed a non-linear response to body weight unloading. DPPA increased at 80% and 60% BW compared with 100% BW, indicating longer contact times; however, at 40% BW, DPPA decreased, suggesting a shortening of contact time.

In contrast, the duration of negative axial acceleration (DPNA) (Table 7) did not change significantly across conditions (*p* > 0.05). Nevertheless, overall contact time increased progressively with higher levels of body weight support. These findings suggest that unloading modifies not only acceleration magnitude but also the temporal distribution of impact-related acceleration characteristics, promoting a more gradual application of forces.

### 3.3. Anterior–Posterior Acceleration Parameters

Significant changes were observed in acceleration components related to propulsion and braking. Posterior acceleration (PNAPA) (Table 8), representing braking forces, decreased significantly with increasing unloading, indicating reduced deceleration demands during foot contact. In parallel, propulsive acceleration (PPAPA) (Table 9) also decreased, suggesting an overall reduction in force generation requirements during forward propulsion. These results indicate that unloading not only reduces impact forces but also alters the mechanical demands of both braking and propulsion phases of running.

### 3.4. Global Loading Parameters

The maximal resultant acceleration (MRA), representing the overall resultant tibial acceleration, did not differ significantly between 100% BW and the moderate unloading conditions of 80% BW and 60% BW. However, a significant reduction in MRA was observed at 40% BW for both the right and left limbs (Table 10). These findings indicate that resultant tibial acceleration was relatively preserved at moderate unloading levels, whereas substantial unloading at 40% BW produced a measurable reduction in global acceleration magnitude.

### 3.5. Comparison Between Conventional Treadmill and 100% BW Condition

An analysis comparing conventional treadmill running and the 100% BW condition on the microgravity treadmill revealed no significant differences across most evaluated parameters, and effect sizes were generally small. However, several left-side variables demonstrated statistically significant differences, including MAA, PPAA, PNAA, DPNA, and PPAPA (Table 11). In these variables, acceleration-related values were slightly lower under the 100% BW microgravity treadmill condition. These findings suggest overall biomechanical similarity between systems, while also indicating that treadmill surface and system characteristics may subtly influence local tibial acceleration measurements even under nominally equivalent body-weight conditions.

### 3.6. Additional Analysis of Side-Related Effects

To verify whether the observed changes differed between limbs, an additional analysis including the factor of body side (left vs. right) was performed. No significant main effect of Side was observed for any of the analyzed parameters (*p* > 0.05). Furthermore, no significant interactions between Side and experimental conditions were identified. Although isolated differences between the left and right tibia were noted in selected comparisons, these were not consistent across conditions and did not indicate a meaningful side-related effect.

## 4. Discussion

Partial body weight support systems and microgravity treadmills are used because they allow mechanical loading to be reduced without completely eliminating running. Some studies indicate that as the level of unloading increases, ground reaction forces, force impulses, cadence, and vertical stiffness decrease, while step time, flight time, and ground contact time increase [14,15,16,17]. At the same time, it is possible to maintain a relatively high metabolic cost by appropriately increasing treadmill speed or incline [16]. It is precisely this combination of reduced mechanical loading with the ability to preserve a training stimulus that constitutes the primary rationale for the clinical use of microgravity treadmills in the rehabilitation of runners [16,21].

The results of our study are consistent with this overall picture, but also refine it, as they refer not to global ground reaction forces but to local tibial acceleration indicators associated with loading exposure. The clearest pattern concerned amplitude-related parameters. Mean axial acceleration (MAA) on the left side decreased from 3.40 on a conventional treadmill to 3.23 at 100% BW, 2.88 at 80% BW, 2.48 at 60% BW, and 1.93 at 40% BW, corresponding to reductions of approximately 5%, 15%, 27%, and 43%, respectively. On the right side, the trend was very similar: 3.30, 3.19, 2.88, 2.48, and 1.92, representing decreases of approximately 4%, 13%, 25%, and 42%. This pattern is biomechanically consistent with the assumption that fluctuations in tibial axial acceleration decrease, and thus exposure to impact-related loading components is also reduced. In clinical practice, this parameter appears to be one of the most useful for monitoring “how much impact-related acceleration exposure has been reduced” from the limb.

A similarly clear decrease was observed for PPAA—the positive peak axial acceleration at heel strike. On the left side, values decreased from 1.89 to 1.83, 1.82, 1.66, and 1.45, and on the right side from 1.86 to 1.84, 1.84, 1.71, and 1.50. This indicates that the transition from a conventional treadmill to microgravity treadmills alone produced only a minor change, whereas clinically meaningful reductions emerged at 60% and especially at 40% of body weight. An even stronger effect was observed for PNAA. On the left side, values decreased from 1.40 to 1.28, 0.92, 0.45, and 0.17, and on the right side from 1.35 to 1.24, 0.91, 0.54, and 0.24. This corresponds to reductions of approximately 8% at 100%, 33–35% at 80%, about 60–68% at 60%, and over 80% at the 40% condition. In practice, this means that the negative peak acceleration associated with the push-off phase is one of the most sensitive indicators of unloading. From a biomechanical perspective, this suggests a reduction in impact-related mechanical exposure rather than a direct quantification of bone loading. It is important to emphasize that tibial acceleration should be interpreted as an indirect proxy of impact loading and not as a direct measure of bone stress. This is important because the literature on tibial acceleration emphasizes that it is a proxy for impact loading rather than a direct measure of bone stress [7,22,23,24]. Sheerin et al. [7] indicate that tibial acceleration is a useful indicator of how technique, speed, limb stiffness, surface, and footwear influence impact exposure, but its relationship with actual bone stress is not straightforward and should not be treated as one-to-one. Moreover, the recorded signal may be influenced by soft tissue artifacts, including the damping properties of skin and adipose tissue, meaning that the measured acceleration does not represent pure bone motion. At the same time, other authors emphasize that multi-axis measurement, rigid sensor fixation, and well-controlled protocols improve its clinical and research value [24,25,26]. The review by Xiang et al. [27] also highlights that the relationship between tibial acceleration, ground reaction forces, and actual bone stress and internal loading conditions is complex and partly mediated by neuromuscular strategies and shock attenuation.

In this context, our results are particularly interesting because, beyond a simple reduction in amplitude, they also demonstrate a change in the temporal distribution of loading. Time to peak positive axial acceleration (TPPAA) on the left side increased from 324 ms on a conventional treadmill to 340 ms at 100% BW, 347 ms at 80% BW, 347 ms at 60% BW, and 358 ms at 40% BW. On the right side, the pattern was less consistent at 100%, but at 60% and 40% the increase was already clear: from 321 ms to 349 and 369 ms. This indicates that with greater unloading, the peak positive acceleration occurs later, meaning that loading not only decreases but is also distributed over a longer time period. Such a pattern can be interpreted as a “softer” ground contact, potentially more favorable for bone tissue and tendon insertions. On the other hand, the parameters DPPA and DPNA did not exhibit such a clear pattern. DPPA on the left side increased at 100% and 60%, but did not increase linearly toward 40%; on the right side, the changes were even less consistent. DPNA also did not follow a simple monotonic trend. This suggests that unloading does not merely “remove force,” but also induces a partial reorganization of locomotor strategy. This conclusion is consistent with previous biomechanical studies [14,18,28]. Vincent et al. [21] summarize that with increasing unloading on a microgravity treadmill, cadence, ground reaction forces, knee and ankle joint ranges of motion, and vertical stiffness decrease, while flight time, contact time, and step length increase. It was demonstrated that natural cadence decreases by approximately 1.5–3.5% for every 10% change in body weight support, with about a 10% reduction expected at around 50% BW. As suggested by these authors, such adaptations may partially compensate for the reduction in loading resulting from unloading [14,18,28]. This is consistent with the concept of lower-limb stiffness regulation and suggests that the actual reduction in acceleration-based indicators associated with bone loading may be smaller than would be expected based solely on reduced body weight [14,18,28]. Our data show a very similar trend. Cadence was unchanged between the conventional treadmill and microgravity treadmill at 100% BW (approximately +0.4%), but decreased by about 4% at 80% BW, 10% at 60% BW, and 17–18% at 40% BW. This is clinically important, as it suggests that 80% BW may still represent a zone of a relatively preserved running pattern, whereas 60% BW and especially 40% BW correspond to levels at which changes in movement strategy become substantial and may partially compensate for or mask the effects of unloading itself. In other words, greater unloading provides a stronger protective effect, but at the cost of progressively deviating from natural running mechanics. This is also evident in MRA—maximal resultant acceleration. On the left side, values were 2.55, 2.52, 2.58, 2.51, and 2.25, and on the right side 2.49, 2.52, 2.57, 2.50, and 2.24. Thus, this global loading indicator decreased clearly only at 40% BW, whereas at 80% BW and partly at 60% BW the pattern was less straightforward. This supports the hypothesis that compensatory strategies emerge at intermediate levels of unloading—most likely related to changes in lower-limb stiffness, flight phase characteristics, braking mechanisms, and propulsion generation. Such behavior is consistent with the concept of self-regulation of musculoskeletal system stiffness under varying surface and loading conditions, as described in the running biomechanics literature [21,29,30].

An important, yet still relatively underexplored issue in treadmill running biomechanics is the influence of the material properties and structural design of the treadmill running surface (i.e., belt composition and construction) on the magnitude of loads acting on the lower limb. In treadmills such as Woodway, a characteristic construction is used based on segmented slats (slat-belt) made of vulcanized rubber, rather than a conventional continuous belt made of synthetic material tensioned over a rigid deck. To date, most studies have focused on comparisons between treadmill and overground running, demonstrating differences in muscle activation, kinematic parameters, and physiological responses [31,32], whereas the specific material properties of treadmill belts (e.g., compliance, cushioning capacity) have rarely been treated as an experimental variable. Huang et al. [30] showed that treadmills equipped with cushioning systems can reduce ground reaction forces by up to approximately 10% compared to standard designs, which may translate into lower loads acting on the knee and ankle joints. At the same time, other studies indicate that more compliant running surfaces may reduce joint loading; however, this effect is often partially compensated for by biomechanical adaptations of the runner, such as changes in lower-limb stiffness or modifications of movement patterns [14,32,33]. This phenomenon is further supported by studies demonstrating that the body actively adjusts musculoskeletal stiffness in response to changes in surface properties, which may lead to relatively stable ground reaction force values despite differences in surface compliance [29].

The comparison between the conventional treadmill and the 100% BW condition on the microgravity treadmill was included to determine whether the treadmill system itself influenced tibial acceleration independently of unloading. This distinction is important because microgravity treadmills differ from conventional treadmills not only in effective body-weight support, but also in belt construction, surface compliance, pressure stabilization, and overall mechanical characteristics. Therefore, establishing a reference condition at nominally equivalent body weight allowed us to better isolate the biomechanical effects associated specifically with unloading. Overall, the results demonstrated broad biomechanical similarity between conditions, as most parameters showed non-significant differences and small effect sizes. However, several left-side variables (MAA, PPAA, PNAA, DPNA, and PPAPA) demonstrated statistically significant differences. Importantly, these differences were unilateral, relatively small in magnitude, and were not accompanied by a significant main effect of Side or Side × Condition interaction in the repeated-measures ANOVA. Therefore, they likely reflect subtle asymmetries related to individual motor strategies, sensor positioning variability, or small differences in treadmill–surface interaction rather than a systematic biomechanical effect of the treadmill system itself. Nevertheless, these findings indicate that the treadmill system may slightly influence local tibial acceleration measurements even under 100% BW conditions. Accordingly, the interpretation of unloading effects should acknowledge that both effective body-weight reduction and treadmill-system characteristics may contribute to the observed biomechanical responses. In the context of local bone loading, available data remain limited; however, they indicate that unloading leads to a reduction in tibial acceleration, which is considered a proxy for impact loading [7,10,33]. Most studies on microgravity treadmills, however, focus on global biomechanical parameters, while the analysis of local bone tissue loading remains insufficient [15,16,17,18]. Additionally, it has been shown that higher levels of unloading (≥40–60% of body weight) lead to significant alterations in running patterns, which may limit the transfer of training effects to real-world conditions [14,31,32]. Therefore, moderate unloading is often recommended as a compromise between reducing mechanical loading and preserving natural movement biomechanics. The results of the present study are consistent with these observations, indicating that progressive unloading reduces loads acting on the tibia, while highlighting that determining the optimal level of unloading remains critical in the context of rehabilitation and safe return to activity.

This leads to an important practical question: which levels of unloading may be clinically optimal? However, it should be emphasized that the present study was conducted in healthy participants, and the following considerations should be interpreted with caution. In the case of tibial stress fractures, Liem et al. [34] suggest initiating running on a microgravity treadmill at approximately 50–65% BW, maintaining this level for at least one week, and then increasing the load by 5–10% BW per week, provided there is no pain during or after sessions. Overground running is proposed once the patient tolerates approximately 85–90% BW [5,34]. Vincent et al. [21] are somewhat more conservative and suggest that a safe transition to overground running is likely when the athlete can run pain-free for more than 30 min at >95% BW. When these recommendations are considered alongside our findings, a tentative, hypothesis-generating three-stage model may be proposed rather than a direct clinical guideline. In the most symptomatic phase, the greatest protective effect in our data was observed at 60% and 40% BW, where reductions in MAA, PNAA, and braking-related parameters were the largest. However, these are also the levels at which the greatest changes in cadence and running pattern occur. Therefore, levels around 60–70% BW may represent a reasonable starting point from a biomechanical perspective, when the primary goal is to reduce local bone loading while maintaining exercise tolerance, but not yet restoring natural mechanics. Subsequently, 80–85% BW may be considered a transitional zone, as we still observed meaningful reductions in axial indicators and negative peak acceleration, while the decrease in cadence was only about 4%. Finally, 85–95% BW may represent a potential pre-return zone, consistent with previous literature [21,34], although this should be confirmed in clinical populations.

In the case of Osgood–Schlatter disease (OSD), the situation is more complex, as there are no direct studies involving microgravity treadmills. Current literature on OSD primarily emphasizes activity modification, pain monitoring, progressive strengthening, and gradual return to sport, rather than a specific “percentage of unloading” on a treadmill. However, recent reviews highlight that high-quality evidence for specific conservative treatment methods remains limited, and many clinical decisions are still based on load management principles [35]. Therefore, any application of microgravity treadmills in this context should be considered exploratory and not evidence-based. Nevertheless, our data allow for a conceptual framework rather than a clinical recommendation. Since OSD affects the tibial tuberosity and proximal structures, it would be desirable to reduce not only axial peak loading but also braking components and rapid changes in acceleration. In our data, 80% BW appears to be an attractive compromise: MAA decreased by approximately 13–15%, PNAA by approximately 33–35%, while cadence decreased by only about 4%. This suggests that a range of 80–90% BW could be considered as a potential option, but only as a hypothesis requiring further validation. Importantly, all of the above considerations should be interpreted as biomechanical insights rather than direct clinical prescriptions. In summary, our results indicate that unloading on the microgravity treadmill effectively reduces tibial acceleration parameters associated with impact-related loading exposure; however, not all parameters respond linearly, and greater levels of unloading increase the likelihood of compensatory changes in running patterns. From a practical perspective, the most promising “compromise range” appears to be around 80% BW: the reduction in key loading indicators is already substantial, while changes in cadence and overall running pattern remain moderate. In tibial stress fractures, more aggressive unloading in the range of 60–70% BW may be beneficial at the beginning of rehabilitation, but should be progressively increased to 85–95% BW before returning to overground running. In Osgood–Schlatter disease, it seems more appropriate to use 80–90% BW as a tool for symptom control while maintaining relatively natural biomechanics, with a possible temporary reduction to 70–80% BW during periods of pain exacerbation. However, these proposals require direct clinical validation, as the current literature remains considerably richer in data on global biomechanical parameters than on local tibial bone loading.

The interpretation of the present study results should take into account some limitations. First, tibial accelerations were used as an indirect proxy of bone loading rather than a direct measurement. As indicated in previous research, the relationship between segment acceleration and actual bone stress is complex and depends, among other factors, on the damping properties of soft tissues, neuromuscular strategies, and movement parameters. Therefore, the obtained results should be interpreted as indicators of relative changes in loading rather than absolute values. Second, accelerometer-based measurements are sensitive to sensor placement and fixation. Despite the use of a standardized protocol and positioning of the sensor near the tibial tuberosity, the influence of soft tissue artifacts and minor differences in sensor orientation between participants cannot be entirely excluded. Third, the microgravity treadmill trials were performed in a fixed order from 100% to 40% BW. This sequence was selected to provide a conservative and well-tolerated transition from full effective body weight to progressively reduced loading, while maintaining identical testing conditions across participants. Anti-gravity treadmill systems allow controlled manipulation of effective body weight and have been used to reduce impact forces while preserving running-specific movement patterns. Therefore, the fixed order was intended to improve participant tolerance and procedural consistency. However, because the order was not randomized, potential order or adaptation effects cannot be excluded; consequently, the findings should be interpreted as differences associated with unloading conditions rather than as definitive causal effects of the progressive sequence itself. Fourth, participants performed all trials in their own running shoes. Although this approach was chosen to preserve natural running patterns and avoid the need for adaptation to unfamiliar footwear, differences in shoe characteristics (e.g., cushioning, stiffness) may have influenced the measured biomechanical parameters. It should also be noted that the study had a cross-sectional design and was conducted under laboratory conditions, which limits the direct generalizability of the findings to real-world overground running.

## 5. Conclusions

The findings of this study indicate that progressive body weight unloading during running on a microgravity treadmill leads to a significant reduction in tibial acceleration parameters associated with impact-related loading exposure, particularly in parameters describing the amplitude and dynamics of axial accelerations. The greatest changes were observed at higher levels of unloading (60% and 40% BW), where reductions in these indicators were most pronounced. At the same time, the relationship between the level of unloading and loading magnitude was not fully linear. Comparison between conventional treadmill running and microgravity treadmill running at 100% BW demonstrated overall biomechanical similarity between conditions; however, several unilateral differences in tibial acceleration parameters suggest that treadmill-system and surface characteristics may exert a subtle influence on local acceleration measurements. The results also indicate that unloading levels around 80% BW may represent a compromise between load reduction and preservation of a relatively natural running pattern. However, these findings should be interpreted with caution, as the study was conducted in healthy participants and does not directly translate to clinical populations. Therefore, any clinical application of these results should be considered preliminary and requires validation in injured populations. This study contributes to the literature by providing data on local tibial acceleration-based biomechanical indicators associated with loading exposure, which may support future research in both clinical and training contexts.

## Figures and Tables

**Table 1:** Cadence across running conditions.

Outcome Measure Cadence (Steps/min)	Side	Mean ± SD (1)	Mean ± SD (2)	*p*	ES
80% BW (1) vs. 100% BW (2)	R	151 ± 8	159 ± 8	<0.001	1.00
	L	151 ± 8	159 ± 8	<0.001	1.00
60% BW (1) vs. 100% BW (2)	R	143 ± 7	159 ± 8	<0.001	2.12
	L	143 ± 7	159 ± 8	<0.001	2.12
40% BW (1) vs. 100% BW (2)	R	129 ± 9	159 ± 8	<0.001	3.52
	L	131 ± 12	159 ± 8	<0.001	2.74

BW—Body Weight; R—Right Side, L—Left Side; SD—Standard Deviation; *p*—*p*-value; ES—effect size.

**Table 2:** Magnitude of Axial Acceleration across running conditions.

Outcome Measure Magnitude of Axial Acceleration (MAA) [g]	Side	Mean ± SD (1)	Mean ± SD (2)	*p*	ES
80% BW (1) vs. 100% BW (2)	R	2.86 ± 0.35	3.19 ± 0.36	<0.001	0.92
	L	2.88 ± 0.28	3.23 ± 0.38	<0.001	1.04
60% BW (1) vs. 100% BW (2)	R	2.47 ± 0.25	3.19 ± 0.36	<0.001	2.32
	L	2.48 ± 0.28	3.23 ± 0.38	<0.001	2.24
40% BW (1) vs. 100% BW (2)	R	1.93 ± 0.35	3.19 ± 0.36	<0.001	3.54
	L	1.92 ± 0.25	3.23 ± 0.38	<0.001	4.07

BW—Body Weight; R—Right Side, L—Left Side; SD—Standard Deviation; *p*—*p*-value; ES—effect size.

**Table 3:** Peak Positive Axial Acceleration across running conditions.

Outcome Measure Peak Positive Axial Acceleration (PPAA) [g]	Side	Mean ± SD (1)	Mean ± SD (2)	*p*	ES
80% BW (1) vs. 100% BW (2)	R	1.82 ± 0.20	1.84 ± 0.21	0.461	0.09
	L	1.82 ± 0.20	1.83 ± 0.21	0.853	0.04
60% BW (1) vs. 100% BW (2)	R	1.71 ± 0.21	1.84 ± 0.21	<0.001	0.61
	L	1.66 ± 0.23	1.83 ± 0.21	<0.001	0.77
40% BW (1) vs. 100% BW (2)	R	1.50 ± 0.23	1.84 ± 0.21	<0.001	1.54
	L	1.45 ± 0.21	1.83 ± 0.21	<0.001	1.80

BW—Body Weight; R—Right Side, L—Left Side; SD—Standard Deviation; *p*—*p*-value; ES—effect size.

**Table 4:** Peak Negative Axial Acceleration across running conditions.

Outcome Measure Peak Negative Axial Acceleration (PNAA) [g]	Side	Mean ± SD (1)	Mean ± SD (2)	*p*	ES
80% BW (1) vs. 100% BW (2)	R	0.90 ± 0.30	1.24 ± 0.31	<0.001	1.11
	L	0.91 ± 0.24	1.28 ± 0.30	<0.001	1.36
60% BW (1) vs. 100% BW (2)	R	0.54 ± 0.17	1.24 ± 0.31	<0.001	2.80
	L	0.45 ± 0.27	1.28 ± 0.30	<0.001	2.90
40% BW (1) vs. 100% BW (2)	R	0.24 ± 0.16	1.24 ± 0.31	<0.001	4.05
	L	0.17 ± 0.21	1.28 ± 0.30	<0.001	4.28

BW—Body Weight; R—Right Side, L—Left Side; SD—Standard Deviation; *p*—*p*-value; ES—effect size.

**Table 5:** Time to Peak Positive Axial Acceleration across running conditions.

Outcome Measure Time to Peak Positive Axial Acceleration (TPPAA) [ms]	Side	Mean ± SD (1)	Mean ± SD (2)	*p*	ES
80% BW (1) vs. 100% BW (2)	R	330 ± 23	321 ± 22	0.114	0.39
	L	347 ± 42	340 ± 44	0.114	0.16
60% BW (1) vs. 100% BW (2)	R	349 ± 22	321 ± 22	<0.001	1.27
	L	347 ± 24	340 ± 44	0.561	0.19
40% BW (1) vs. 100% BW (2)	R	369 ± 29	321 ± 22	<0.001	1.86
	L	358 ± 33	340 ± 44	0.140	0.46

BW—Body Weight; R—Right Side, L—Left Side; SD—Standard Deviation; *p*—*p*-value; ES—effect size.

**Table 6:** Duration of Positive Axial Acceleration across running conditions.

Outcome Measure Duration of Positive Axial Acceleration (DPPA) [%]	Side	Mean ± SD (1)	Mean ± SD (2)	*p*	ES
80% BW (1) vs. 100% BW (2)	R	64 ± 14	60 ± 14	0.166	0.28
	L	54 ± 20	56 ± 15	0.648	0.11
60% BW (1) vs. 100% BW (2)	R	67 ± 21	60 ± 14	0.278	0.39
	L	61 ± 22	56 ± 15	0.448	0.26
40% BW (1) vs. 100% BW (2)	R	55 ± 40	60 ± 14	0.667	0.16
	L	52 ± 38	56 ± 15	0.727	0.13

BW—Body Weight; R—Right Side, L—Left Side; SD—Standard Deviation; *p*—*p*-value; ES—effect size.

**Table 7:** Duration of Negative Axial Acceleration across running conditions.

Outcome Measure Duration of Negative Axial Acceleration (DPNA) [%]	Side	Mean ± SD (1)	Mean ± SD (2)	*p*	ES
80% BW (1) vs. 100% BW (2)	R	4.11 ± 1.42	3.93 ± 1.79	0.665	0.11
	L	3.62 ± 2.03	4.10 ± 1.77	0.192	0.25
60% BW (1) vs. 100% BW (2)	R	2.91 ± 1.86	3.93 ± 1.79	0.055	0.55
	L	4.47 ± 4.22	4.10 ± 1.77	0.757	0.11
40% BW (1) vs. 100% BW (2)	R	4.19 ± 4.34	3.93 ± 1.79	0.806	0.07
	L	5.66 ± 5.32	4.10 ± 1.77	0.291	0.39

BW—Body Weight; R—Right Side, L—Left Side; SD—Standard Deviation; *p*—*p*-value; ES—effect size.

**Table 8:** Peak Positive Anterior–Posterior Acceleration across running conditions.

Outcome Measure Peak Positive Anterior–Posterior Acceleration (PPAPA) [g]	Side	Mean ± SD (1)	Mean ± SD (2)	*p*	ES
80% BW (1) vs. 100% BW (2)	R	0.08 ± 0.29	0.13 ± 0.37	0.135	0.15
	L	0.01 ± 0.30	0.05 ± 0.45	0.358	0.10
60% BW (1) vs. 100% BW (2)	R	−0.24 ± 0.30	0.13 ± 0.37	<0.001	0.32
	L	−0.20 ± 0.20	0.05 ± 0.45	<0.001	0.43
40% BW (1) vs. 100% BW (2)	R	−0.44 ± 0.29	0.13 ± 0.37	<0.001	0.93
	L	−0.49 ± 0.18	0.05 ± 0.45	<0.001	1.28

BW—Body Weight; R—Right Side, L—Left Side; SD—Standard Deviation; *p*—*p*-value; ES—effect size.

**Table 9:** Peak Negative Anterior–Posterior Acceleration across running conditions.

Outcome Measure Peak Negative Anterior–Posterior Acceleration (PNAPA) [g]	Side	Mean ± SD (1)	Mean ± SD (2)	*p*	ES
80% BW (1) vs. 100% BW (2)	R	−1.77 ± 0.14	−1.90 ± 0.06	0.007	1.20
	L	−1.80 ± 0.10	−1.88 ± 0.09	0.030	0.84
60% BW (1) vs. 100% BW (2)	R	−1.57 ± 0.20	−1.90 ± 0.06	<0.001	2.23
	L	−1.60 ± 0.19	−1.88 ± 0.09	<0.001	1.88
40% BW (1) vs. 100% BW (2)	R	−1.31 ± 0.24	−1.90 ± 0.06	<0.001	3.37
	L	−1.39 ± 0.22	−1.88 ± 0.09	<0.001	3.29

BW—Body Weight; R—Right Side, L—Left Side; SD—Standard Deviation; *p*—*p*-value; ES—effect size.

**Table 10:** Maximal Resultant Acceleration across running conditions.

Outcome Measure Maximal Resultant Acceleration (MRA) [g]	Side	Mean ± SD (1)	Mean ± SD (2)	*p*	ES
80% BW (1) vs. 100% BW (2)	R	2.57 ± 0.22	2.52 ± 0.24	0.323	0.21
	L	2.58 ± 0.24	2.51 ± 0.24	0.166	0.29
60% BW (1) vs. 100% BW (2)	R	2.50 ± 0.21	2.52 ± 0.24	0.683	0.08
	L	2.51 ± 0.28	2.51 ± 0.24	0.954	0.11
40% BW (1) vs. 100% BW (2)	R	2.24 ± 0.35	2.52 ± 0.24	0.016	0.93
	L	2.25 ± 0.23	2.51 ± 0.24	0.005	1.10

BW—Body Weight; R—Right Side, L—Left Side; SD—Standard Deviation; *p*—*p*-value; ES—effect size.

**Table 11:** Comparison Between Conventional Treadmill and 100% BW Condition.

Outcome Measure	Side	Mean ± SD (1) Conventional Treadmill	Mean ± SD (2) 100% BW	*p*	ES
Cadence (steps/min)	R	158 ± 8	159 ± 8	0.600	0.12
	L	158 ± 8	159 ± 8	0.636	0.12
Magnitude of Axial Acceleration (MAA) [g]	R	3.30 ± 0.37	3.19 ± 0.36	0.188	0.30
	L	3.40 ± 0.42	3.23 ± 0.38	0.020	0.30
Peak Positive Axial Acceleration (PPAA) [g]	R	1.86 ± 0.15	1.84 ± 0.21	0.461	0.10
	L	1.89 ± 0.13	1.83 ± 0.21	0.031	0.10
Peak Negative Axial Acceleration (PNAA) [g]	R	1.35 ± 0.32	1.24 ± 0.31	0.124	0.34
	L	1.40 ± 0.37	1.28 ± 0.30	0.011	0.35
Time to Peak Positive Axial Acceleration (TPPAA) [ms]	R	326 ± 37	321 ± 22	0.345	0.16
	L	324 ± 38	340 ± 44	0.256	0.38
Duration of Positive Axial Acceleration (DPPA) [%]	R	60 ± 15	60 ± 14	0.838	0.11
	L	52 ± 11	56 ± 15	0.227	0.30
Duration of Negative Axial Acceleration (DPNA) [%]	R	3.47 ± 1.85	3.93 ± 1.79	0.244	0.25
	L	3.30 ± 0.89	4.10 ± 1.77	0.035	0.57
Peak Positive Anterior–Posterior Acceleration (PPAPA) [g]	R	0.19 ± 0.45	0.13 ± 0.37	0.602	0.14
	L	0.19 ± 0.43	0.05 ± 0.45	0.015	0.32
Peak Negative Anterior–Posterior Acceleration (PNAPA) [g]	R	−1.87 ± 0.10	−1.90 ± 0.06	0.269	0.36
	L	−1.90 ± 0.06	−1.88 ± 0.09	0.591	0.26
Maximal Resultant Acceleration (MRA) [g]	R	2.49 ± 0.30	2.52 ± 0.24	0.448	0.11
	L	2.55 ± 0.22	2.51 ± 0.24	0.335	0.13

BW—Body Weight; R—Right Side, L—Left Side; SD—Standard Deviation; *p*—*p*-value; ES—effect size.

## Data Availability

All data generated or analyzed during this study are included in this published article.
